# Airborne Visual Detection and Tracking of Cooperative UAVs Exploiting Deep Learning

**DOI:** 10.3390/s19194332

**Published:** 2019-10-07

**Authors:** Roberto Opromolla, Giuseppe Inchingolo, Giancarmine Fasano

**Affiliations:** Department of Industrial Engineering, University of Naples Federico II, Piazzale Tecchio 80, 80125 Naples, Italy; giu.inch@gmail.com (G.I.); giancarmine.fasano@unina.it (G.F.)

**Keywords:** unmanned aerial vehicles, UAV swarms, visual detection, visual tracking, machine vision, deep learning, YOLO

## Abstract

The performance achievable by using Unmanned Aerial Vehicles (UAVs) for a large variety of civil and military applications, as well as the extent of applicable mission scenarios, can significantly benefit from the exploitation of formations of vehicles able to fly in a coordinated manner (swarms). In this respect, visual cameras represent a key instrument to enable coordination by giving each UAV the capability to visually monitor the other members of the formation. Hence, a related technological challenge is the development of robust solutions to detect and track cooperative targets through a sequence of frames. In this framework, this paper proposes an innovative approach to carry out this task based on deep learning. Specifically, the You Only Look Once (YOLO) object detection system is integrated within an original processing architecture in which the machine-vision algorithms are aided by navigation hints available thanks to the cooperative nature of the formation. An experimental flight test campaign, involving formations of two multirotor UAVs, is conducted to collect a database of images suitable to assess the performance of the proposed approach. Results demonstrate high-level accuracy, and robustness against challenging conditions in terms of illumination, background and target-range variability.

## 1. Introduction

Nowadays, Unmanned Aerial Vehicles (UAVs) represent a reliable and affordable tool suitable for many applications, such as intelligence, surveillance and reconnaissance (ISR) [1], aerial photography [2], infrastructure monitoring [3], search and rescue [4], and precision agriculture [5]. Indeed, the exponential growth in the use of these vehicles has been driven, in recent years, by the miniaturization and cost reduction of electronic components (e.g., microprocessors, sensors, batteries and wireless communication units) which have supported the development of small and micro UAVs [6]. However, while these systems have demonstrated notable advantages being cheap, flexible and easy to deploy, they still have limitations in practical scenarios due to their lower payload capacity, shorter endurance and operational range. These aspects have led to the concept of UAV swarms, i.e., formations of UAVs able to fly in a coordinated manner to provide increased mission performance, reliability, robustness and scalability while ensuring a reduced mission cost [7,8]. For instance, the coordinated flight of multiple UAVs can allow faster area coverage [9,10], improve navigation performance under nominal GNSS coverage [11,12], or enable safe flight in GNSS-challenging areas [13,14].

Clearly, some key challenges must be addressed to unleash the full potential of UAV swarms, thus achieving performance improvement, with respect to missions carried out by standalone UAVs. These challenges may include, but are not limited to, cooperative guidance/control, communication, navigation and situational awareness. In view of guidance and control issues, UAV swarms can act as either centralized or decentralized networks depending on whether the main processing and decision-making functions are concentrated on a single node of the network or distributed among all the nodes [15,16]. Communication issues are also important, and different solutions may be exploited to ensure adequate UAV-UAV datalink, such as Wi-Fi, Ultra/Very High Frequency, LTE, Satcom or based on airborne transponder [17,18], where the choice of the most convenient strategy depends on factors like application scenario, cost budgets and types of UAV involved (e.g., small vs. large UAVs). Finally, regarding navigation and situational awareness, collaborative and relative sensing issues may arise. Collaborative sensing is the problem of exploiting onboard sensors and processing resources in a cooperative way to obtain specific goals, like object detection and tracking, surveillance and data collection [19]. Instead, relative sensing is aimed at getting information about the relative and absolute state of multiple UAVs, and it can have critical importance to enable safe control of the formation or to improve navigation performance when the formation operates in complex environments [20,21].

This paper focuses its attention on relative sensing aspects. Indeed, in some mission scenarios, it may be extremely important that each UAV is able to detect and track the other members of the swarm obtaining information such as relative distance and bearing (Line-of-Sight, LOS). Considering the cooperative nature of the formation, this task can be carried out using various solutions based on acoustic sensors, Radio-Frequency ranging, LIDAR and visual cameras [22,23,24,25]. In this respect, despite their limited applicability in absence of adequate illumination, visual cameras are extremely versatile sensors, being low-cost, lightweight and able to provide highly-accurate LOS estimates. Clearly, this latter aspect is achieved if image processing algorithms, able to determine the target position in the image plane with pixel-level accuracy, are developed. This goal involves complex technical challenges. Some of the most important are listed below:

- *Sun illumination*. Indeed, wide portions of the field-of-view (FOV) may be saturated if the Sun direction is quasi-frontal with respect to the camera. Also, the object appearance (e.g., color and intensity) can change as a function of the Sun direction.
- *Local background*. Detection and tracking algorithms may suffer from sudden variations of the local background, e.g., caused by continuous passages of the target above/below the horizon.
- *Target scale*. Depending on the mission scenario, the target distance can vary from tens to hundreds of meters. Consequently, the image processing algorithms must be able to deal with significant variations of the size of FOV region occupied by the target.

In this framework, artificial intelligence has recently produced a breakthrough in the computer vision field, especially due to the advancements of deep learning techniques [26,27]. Hence, this paper aims to analyze the potential of such approaches for airborne visual detection and tracking of cooperative UAVs. Specifically, an original image processing architecture is developed, which exploits the You Only Look Once (YOLO) object detection system [28] as main processing block. A peculiar characteristic of this method is that machine-vision algorithms are aided by navigation data (e.g., own-ship attitude and target distance) some of which are available thanks to the cooperative nature of the formation (e.g., exchanged through a communication link). Validation and performance assessment of the proposed algorithmic architecture are carried out using a database of images collected by means of an experimental campaign carried out using formations of two multirotor UAVs, which are indicated in the following as tracker (the one with the camera on board) and target.

The remainder of the paper is organized as follows. Section 2 reviews the state of the art regarding techniques for airborne visual detection and tracking of flying vehicles, and it highlights the contribution of this work. Section 3 describes in detail the proposed image processing architecture. Section 4 presents the experimental setups and the flight-test data collected for training the deep neural network, and for testing of the overall detection and tracking architecture. Section 5 details the achieved results based on ad-hoc defined performance metrics. Finally, Section 6 gives the conclusions, while also discussing lessons learnt and providing indications about future work.

## 2. Related Work

In the applications of interest to this work, the tracker and the target are both flying vehicles. Thus, extremely fast variations of the target appearance may occur in the image plane. This aspect must be considered in the design of image processing algorithms for airborne detection and tracking of a moving target. An overview of state-of-the-art approaches from the open literature is provided in the following. The main classification criterion is related to the fact that the target UAV can be either cooperative or non-cooperative.

The issues of visual detection and tracking of non-cooperative target UAVs are mainly treated in the frame of Sense and Avoid (SAA) [29,30,31,32,33] and counter-drone applications [34,35]. In SAA scenarios, the main requirement is to maximize the declaration range (i.e., the range at which a firm track of the target is generated) to satisfy safety margins regarding the time needed to perform an avoidance maneuver [29]. Consequently, most research efforts are addressed to very long-range scenarios, where a target occupies only very few pixels in the image plane. The detection task may be carried out exploiting different approaches. Image processing architectures based on the morphological filtering operator are mainly proposed [30,31]. However, intruders may also be detected by computing the difference between two subsequent images (corrected thanks to an homography estimation based on corner features extracted under the horizon) [32]. More recently, the deep learning concept has also been applied to this task by training a Convolutional Neural Network (CNN), and combining this detector with a Bottom-Hat morphological filter [33]. A known issue for standalone optical detection systems is the average number of false detections. This problem is typically addressed by filtering architectures (e.g., Extended Kalman Filters or Particle Filters) designed to generate target tracks from multiple subsequent detections. Recently, the Deep Learning concept has also been applied for counter-drone applications, i.e., to detect intruder, non-cooperative UAVs using one or multiple visual cameras fixed on ground [34,35].

With regards to visual detection and tracking of cooperative UAVs, the interval of relative distances can vary significantly during operation. Hence, the target can either occupy a few pixels or a wide portion of the image plane. While this is certainly a challenge if the range variations occur fast enough, on the other side it gives the possibility to exploits a wider range of image processing approaches, which can be mainly classified into direct- and feature-based methods. Direct-based methods exploit pixel-wise estimation of the local gradient of the image intensity to treat the detection process as the problem to estimate the transformation (registration) which aligns two frames in the best way, i.e., based on a purposely defined error metric function [36]. Instead, feature-based methods exploit the extraction of salient features and the use of proper descriptors to recognize the portion of the image containing the target [37]. Techniques adopting the machine-learning or deep-learning concepts can also be put under this category since, the trained network can be interpreted as a detector and classifier of features [38]. With specific attention to approaches exploited to detect UAVs with airborne cameras the following works can be mentioned. A trained cascade classifier designed for detection and distance estimation of a small quadrotor UAV can be found in [37]. Experimental tests, both outdoor and indoor, show satisfactory performance and demonstrate real-time operation but the method is designed to deal only with relatively-short distances (i.e., below 25 m). The vision-based architecture presented in [39] is designed to detect and track multiple target UAVs using a camera on board a custom, delta-wing, small UAV. First, the background motion is estimated using a perspective transformation model. Then, corner features are extracted from the background-less image and moving objects are detected using optical flow. Though the method is designed to be robust against false alarms, it suffers missed detections for targets below the horizon. A motion-compensation-based approach for airborne detection of UAVs is presented in [40]. Specifically, the main idea is that the detection process should exploit both appearance and motion cues. This is done by combining deep learning (based on a CNN) with an original motion compensation method, both applied over a sequence of frames (namely a spatio-temporal cube). This approach shows better performance than methods based only on either machine learning or motion compensation, but it is better tailored to detect flying vehicles at long range (e.g., for collision avoidance purposes). An object detection and tracking approach which integrates a salient object detector within the Kalman filtering framework is proposed in [41]. Though, it shows better tracking performance than state-of-the-art methods, the authors highlight limitations in dealing with detection of flying targets when placed against a cluttered background. Finally, a recent deep learning based approach, proposed for visual detection and tracking of a drogue object at short range, in the frame of UAV aerial refueling, can be found in [42]. Though some of the abovementioned methods exploit the knowledge of the target, they do not take full advantage of the cooperative nature of the formation. This can be done installing on board artificial markers, such as active Light Emission Diodes (LEDs) [43] and passive retro reflectors [44] or exploiting color-based information if the target has a highly-distinguishable color signature [45,46]. Some limits can be highlighted for these solutions. Specifically, the use of active LEDs involves an increase in the power and weight requirements for the target UAV; passive markers are visible only at relatively-short distances; color-based methods can be negatively affected by illumination conditions which may cause a variation in the color signature. To cope with these issues, machine-vision algorithms could be aided using absolute and relative navigation data, as proposed in [47,48], where the image processing function is based on the combination of template matching and morphological filtering concepts.

The visual detection and tracking approach presented in this work recalls this idea, but navigation data available on board the tracker UAV and cooperative navigation information received from the target UAV are used to aid an original image processing architecture which relies on the deep-learning concept. Specifically, the use of a deep-learning approach is motivated by the need to improve performance (i.e., minimize missed detections without increasing false alarms) when the background is extremely cluttered (e.g., below the horizon) with respect to other machine vision approaches. The innovative points are related to both detection and tracking and are conceived to improve LOS estimation accuracy and ensure adequate robustness against the challenges caused by Sun illumination, local background and target scale. The former aspect, which is extremely important in cooperative applications, is entrusted to an original technique to refine the location of the bounding box which identifies the estimated target position with respect to the one detected by a state-of-the-art DL-based object detector (i.e., YOLO in this work). Regarding the robustness aspect, the use of navigation data not only enables the proposed approach to deal with an extremely wide interval of target-tracker distances, but, as in [47,48], it also allows reducing the occurrence of false alarms and the computational effort thanks to the possibility to focus the target search process over a limited portion of the image plane both during detection and tracking.

## 3. Detection and Tracking Architecture

**Figure 1:**
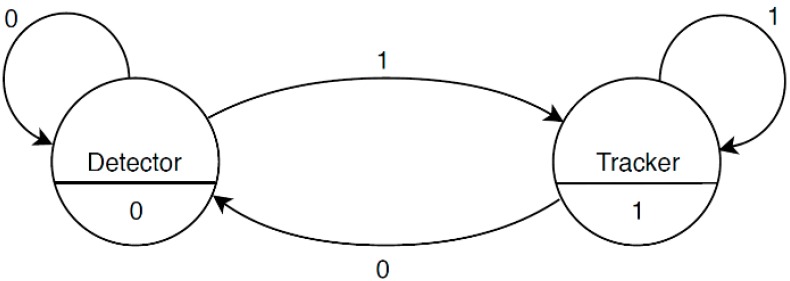
State diagram of the proposed architecture. Two cases are possible for the state of the system, namely target detected (1) or not detected (0). This relatively simple architecture is justified by the cooperative nature of the assumed multi-UAV system.

The proposed architecture is composed of two processing blocks, namely a detector and a tracker, whose goal is to estimate the position of the target UAV on the focal plane of the camera installed on board of the tracker UAV. In the following, the region of the image plane occupied by the target is defined as a rectangular bounding box (BB), from which the unit vector representing the target LOS in the Camera Reference Frame (CRF) can be derived as the direction corresponding to the geometric center of the BB. A state diagram describing this architecture is shown in Figure 1.

The state of the system can be 0 or 1 depending on whether the target position on the image plane at the previous frame is available or not. In the former case, the target identification process is entrusted to the detector which cannot use visual information from the previous frame. As soon as a detection is confirmed, the state becomes 1 and the identification process is entrusted to the tracker, which exploits the knowledge of the target position on the image plane at the previous frame. Clearly, if the tracker fails at detecting the target (missed detection) or if the target is outside the camera FOV, the state of the system returns to 0 and the identification process must be re-initialized by the detector. In the following, the algorithms implemented within the detector and tracking blocks are described in detail. Both these algorithms take advantage of navigation data (e.g., own-ship attitude and relative positioning) to obtain a reliable prediction of the target position on the image plane at the current frame.

### 3.1. DL-Based Detector

**Figure 2:**
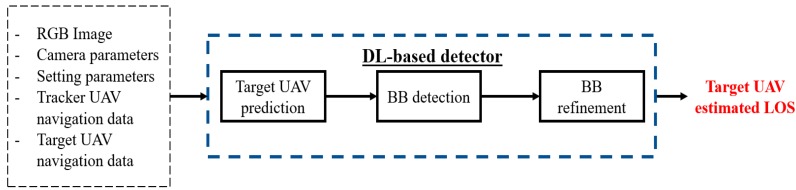
Scheme summarizing the algorithmic strategy characterizing the proposed DL-based detector. The input parameters are listed within a black (dashed) rectangular box. The processing blocks are enclosed within black rectangular boxes. The final output is highlighted in red.

In case of first frame acquisition, or if no information about the target position in the image plane is available from the previous frame, the target search is carried out by the detector block, which exploits the algorithmic strategy whose main steps, inputs and output are highlighted in Figure 2.

First, the *Target UAV prediction* block is used to generate a tentative solution for the target UAV projection on the image plane. Then, the main processing block (*BB detection*) uses this prediction to identify a limited portion of the image plane within which the search for the BB containing the target is carried out exploiting the YOLO object detection system [28]. The BB produced in output by the *BB detection* block is finally used by the *BB refinement* block to obtain a more accurate estimate of target UAV LOS. Detailed information about each of these functions is provided in the following sub-sections.

#### 3.1.1. Target UAV Prediction

This processing step takes advantage of absolute navigation data of the tracker UAV, as well as of target-tracker relative navigation data to predict the projection of the target UAV in the image plane. The relative navigation data are available thanks to the cooperative nature of the proposed visual architecture. Indeed, if a reliable communication link is used to transmit absolute navigation data from the target to the tracker, their relative position vector in the Earth Centered Earth Fixed (ECEF) reference frame (*ρ_e_*) and, consequently, their relative distance (*ρ*) can be estimated. Specifically, this relative positioning information can be obtained with different strategies.

- Computing the difference between the two position vectors in ECEF (*P_e,tracker_* and *P_e,target_* for the tracker and target, respectively), obtained using either the GNSS position fix or, more in general, the positioning solution available to the onboard autopilot.
- Exploiting Differential GNSS (DGNSS) and Carrier-Phase DGNSS (CDGNSS) algorithms.

The choice of the method, which clearly has an impact on the accuracy level of the relative position estimate, depends on the quality of local GNSS coverage for the two vehicles as well as on the type of exchanged data (since differential and carrier-phase differential algorithms require pseudo-range and carrier-phase measurements). Regarding the results presented in this paper, the former approach is exploited to obtain the target-tracker range and relative position vector.

Independently of the adopted solution for relative positioning, the estimated value of *ρ_e_* allows obtaining a prediction of the target-tracker relative position vector in CRF (*ρ_c_*) using Equation (1):

(1) $${\underline{\rho }}_{c}={\underline{\underline{R}}}_{bc}\left({\underline{\underline{R}}}_{nb}{\underline{\underline{R}}}_{en}{\underline{\rho }}_{e}+{\underline{t}}_{cb}\right)$$

where *R_en_* is the rotation matrix from ECEF to the local North-East-Down reference frame (NED), *R_nb_* is the rotation matrix from NED to the Body Reference Frame (BRF) of the tracker UAV, while *R_bc_* and *t_cb_* are the rotation matrix from BRF to CRF and the camera-to-body relative position vector expressed in BRF, respectively. *R_en_* and *R_nb_* can be obtained using the onboard navigation data of the tracker UAV. Specifically, the former depends on the position in ECEF of the tracker UAV, while the latter is an attitude rotation matrix which is also typically parametrized by a 321 sequence of Euler angles (i.e., heading, pitch and roll). Finally, *R_bc_* and *t_bc_* depend on the camera mounting geometry, and they can be computed by carrying out an ad-hoc extrinsic calibration procedure either off-line [49] or on-line [50]. After computing *ρ_c_*, the horizontal (*u_pr_*) and vertical (*v_pr_*) pixel coordinates of the target projection on the image plane can be derived using the intrinsic calibration parameters, i.e., focal length, principal point, skew coefficient and distortion (radial and tangential) coefficients, typically computed carrying out an off-line calibration procedure [51].

#### 3.1.2. Bounding Box Detection

Recently, the problem of using Deep Learning algorithms to address computer vision applications has been deeply investigated [52]. In this framework, several object detection systems based on Deep Learning have been developed, such as the Single Shot MultiBox Detector (SSD) [53], Faster R-CNN [54], RefineDet [55], RFBNet [56] and YOLO [28,57,58]. A detailed overview of object detection systems using Deep Learning including comparisons in terms of accuracy and efficiency can be found in [59,60].

Within the proposed architecture, the target detection task is entrusted to a state-of-the-art object detector, i.e., the YOLO v2 neural network [57]. This DL-based network is obtained using the MATLAB Deep Learning Toolbox [61] (MathWorks, Natick, Massachusetts, USA) starting from a pre-trained CNN, i.e., ResNet-50 [62]. Given a dataset of RGB images taken with the target UAV at different distances relative to the tracker, the training dataset is obtained by randomly cropping each of these images around the target position. Specifically, the cropped image region used for training is a square of 150-by-150 pixels. 

**Figure 3:**
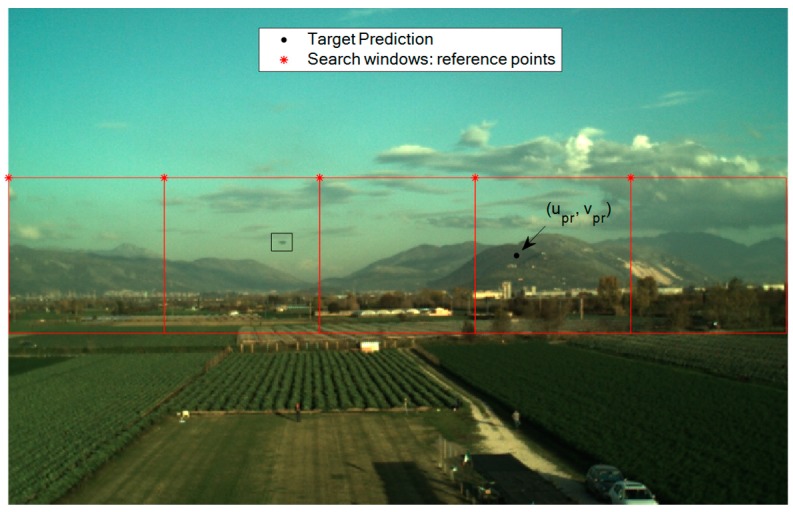
DL-based detector: example of search windows definition for a 752 × 480-pixels RGB image. *d_u_* is set to 150 pixels (*N_w_* = 5). The target UAV position is highlighted by a black box.

**Figure 4:**
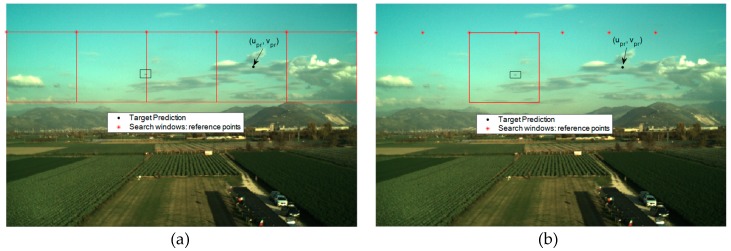
DL-based detector: example of search windows definition for a 752 × 480-pixels RGB image. The target UAV position is highlighted by a black box. (**a**) *d_u_* is set to 150 pixels (*N_w_* = 5). The target UAV projection on the image plane is cut by the border between the second and third search window. (**b**) *d_u_* is set to 100 pixels (*N_w_* = 7). Only the third search window, which fully contains the target UAV, is highlighted for the sake of clarity.

By taking advantage of the predicted target projection obtained at the previous processing step, it is possible to restrain the portion of the image plane to which the trained detector must be applied. Clearly, the shape and the size of the search area should be set considering the uncertainty characterizing each term contributing to the application of Equation (1), i.e., the positions of both the two UAVs, and the attitude of the tracker. Focusing on the uncertainty in the attitude parameters, it is important to highlight that the heading, pitch and roll estimates do not have the same accuracy level. Specifically, due to the possibility to exploit gravity direction, considering performance of low-cost IMUs on board of small UAVs, the uncertainties in the roll and pitch estimates provided by the onboard autopilot are typically bounded at the degree-level [63], while the error in the heading estimate can be significantly larger (up to several degrees) [64]. For this reason, the target search can be limited to a horizontal stripe in the image plane. Of course, the size of the stripe, in general, must account for UAV attitude dynamics, camera frame rate and synchronization uncertainties. In this framework, recent results from our previous work show that if the size of the stripe (*v_max_*) is at least 100 pixels, it is possible to minimize the possibility to accidently remove the target from the analyzed search area [48]. Considering that the YOLO v2 network requires an input RGB image at least as large as the training data, *v_max_* is here set to 150 pixels. So, the defined horizontal stripe can be subdivided into squared regions (search windows) having the same size as the training datasets (150 × 150 pixels), as shown in Figure 3. The reference point used to define each search window is the upper-left corner. The horizontal separation (*d_u_*) between two consecutive search windows, i.e., the distance between two consecutive reference points, is a setting parameter for the user. Clearly, the maximum allowable value of *d_u_* is 150 pixels, since it avoids losing FOV coverage. This value allows minimizing the number of search windows (*N_w_*), and consequently, the algorithm’s computational effort. However, it can be convenient to slightly reduce *d_u_*, thus having a partial overlap between consecutive search windows. Although this solution causes a slight increase in the computational effort (due to the increase in *N_w_*), it also limits the risk that the target is cut by the border of two consecutive search windows as shown, for instance, in Figure 4a. If this occurs, the YOLO v2 detector cannot find “good-quality” BBs, i.e., characterized by large overlap with the target actual position, thus compromising the probability of correct detection. So, a value lower than 150 pixels can be set to *d_u_* to ensure that at least one search window fully contains the target UAV, as shown, for instance, in Figure 4b where *d_u_* is 100 pixels.

Once the target search area is defined, the YOLO v2 detector is applied to each search window independently. This operation produces a large set of BBs representing candidate image regions potentially containing the target according to a specific level of confidence (*S*), i.e., the score measuring the accuracy of the BB. Specifically, the score encodes both the probability that the class of the target object appears in the box and how well the predicted box fits the object [28]. At this point, if the maximum value of *S* (*S_max_*) is higher than a detection threshold (*τ_det_*), to be assigned by the user, the detection process is deemed successful (i.e., the state of the system becomes 1 according to the diagram in Figure 1) and the corresponding BB is sent to the *BB refinement* block.

#### 3.1.3. Bounding Box Refinement

The reasoning behind the introduction of this processing block is that, despite the BB selected at the previous stage may indeed contain the target, there may be a horizontal and/or vertical offset between the center of this BB (from which the target LOS can be estimated knowing the intrinsic calibration parameters of the camera) and the actual center of the target. Such situations tend to degrade the angular accuracy in the estimated target LOS which is extremely important in several tasks involving cooperative UAVs, e.g., to enable highly-accurate cooperative navigation solutions [11].

**Figure 5:**
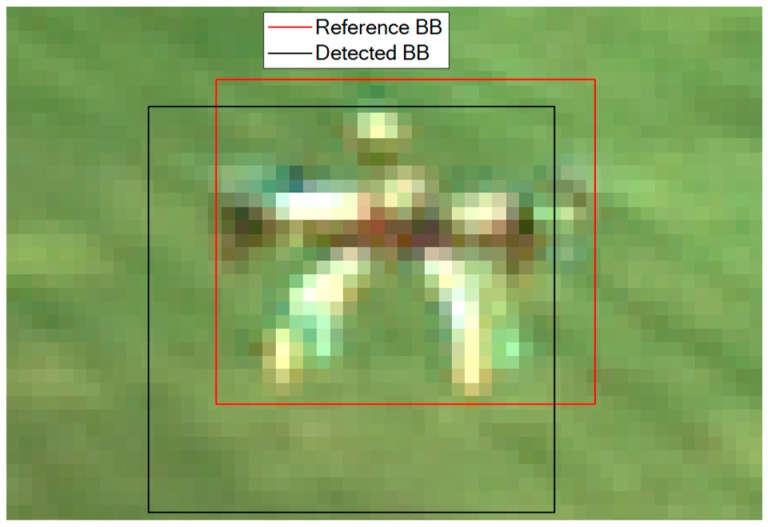
Example of best YOLO detection. *IoU* = 0.538. The reference BB, obtained using a supervised approach, is approximately centered at the geometric center of the target. The detected BB is the output of the DL-based detector.

This phenomenon can also be interpreted as a relatively-poor overlap between the estimated BB and a reference BB (i.e. the one representing the ground truth). The quality of this overlap can be measured using the Intersection over Union (*IOU*) as performance metric. An example is shown in Figure 5, where the *IOU* between the detected and reference BB is 0.538.

**Figure 6:**
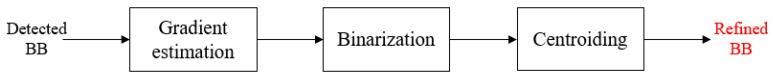
Main steps of the image processing approach to refine the detected BB. An image crop is obtained from the detected bounding box. The gradient operator is applied within this image portion and the gradient image is then binarized. Finally, the centroid of the set of pixels highlighted in the binarized image is computed and a refined BB is centered around this point.

In this case, the estimated position of the target UAV (i.e., the center of the detected BB) is characterized by an offset of about 5 pixels, in both the horizontal and vertical directions. This does not allow taking full advantage of the camera angular resolution, since the errors in the azimuth and elevation angles (see Section 5.1 for details on the estimation of these angles) corresponding to the target LOS in CRF, i.e., 0.21° and 0.26°, respectively are quite larger than the camera IFOV (0.05°). Hence, an approach based on standard machine vision tools is applied to update the BB, whose main steps are highlighted in Figure 6. 

The input can be either the detected BB or an enlarged image portion, i.e., centered at the BB center but with height and width increased by a factor *c* (e.g., between 1.5 and 2). This latter option is useful to avoid the risk that the target is not fully contained in the analyzed area due to the limited overlap of the BB.

**Figure 7:**
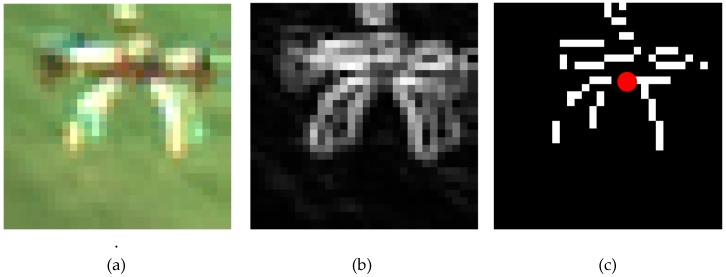
Example of application of the *BB refinement* block. In this case, the factor *c* is 1. (**a**) Detected BB. (**b**) Result of gradient estimation. (**c**) Result of binarization and centroid calculation (highlighted by a red dot).

**Figure 8:**
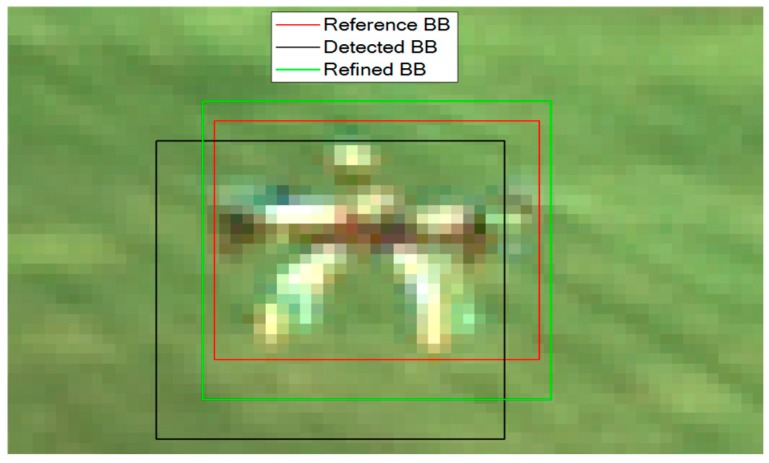
Result of the *BB refinement* algorithm. The *IoU* of the refined BB is 0.747.

An example of application of this procedure is provided by Figure 7. First, the local intensity gradient is computed using the Sobel operator. The resulting gradient image, in which the target is highlighted with respect to the background, is then binarized. Finally, the centroid of the resulting binary image is computed. By centering the refined BB at this point, as shown in Figure 8, it is possible to increase the *IoU* up to 0.747, and the angular errors in the azimuth and elevation angles obtained from the target position on the image plane become 6·10^−4^° and 0.05°, respectively. Considering that the reference BB, i.e., the ground truth (which is obtained manually by a human operator), has an inherent sub-pixel level uncertainty, the very low value obtained for the azimuth error (6·10^−4^°) implies that the refinement process can achieve sub-pixel accuracy (in the horizontal direction only in this case).

### 3.2. DL-Based Tracker

If a detection is confirmed (according to the thresholding criterion presented in Section 3.1.2), the process of searching for the target projection on the image plane of a new frame, is entrusted to a DL-based tracker function. The proposed algorithmic strategy is composed of the same processing blocks presented and described for the DL-based detector. However, consistent differences are relevant to the *Target UAV prediction* and the *BB detection* blocks, mainly due to the possibility to exploit the prior information about the target projection on the image plane available at the previous frame. Specifically, if *ρ_c,los(k-1)_* is the unit vector representing the LOS of the target UAV in CRF estimated at the k-1^th^ frame, the predicted LOS at the current (k^th^) frame (*ρ_c,los(k)_*) can be computed as follows:

(2) $${\underline{\rho }}_{c,los(\text{k})}={\underline{\underline{R}}}_{bc}{\underline{\underline{R}}}_{nb}{}^{k}\left({\underline{\underline{R}}}_{bn}{}^{k-1}{\underline{\underline{R}}}_{cb}{\underline{\rho }}_{c,los(\text{k}-1)}\right)$$

where ${\underline{\underline{R}}}_{nb}{}^{k-1}$ and ${\underline{\underline{R}}}_{nb}{}^{k}$ are the attitude rotation matrixes for the tracker UAV computed by its onboard navigation system. This relation produces highly accurate predictions of the LOS of the target UAV at the current frame provided that the target-tracker relative dynamics is smooth while the frame rate is adequately high, so that the variation of the relative unit vector in NED between two successive frames is negligible. In general, when dealing with particularly aggressive relative dynamics, a more accurate prediction can be obtained by using the velocities of the two UAVs estimated by the corresponding onboard navigation systems.

The result of Equation (2) can then be used to estimate *u_pr_* and *v_pr_* using the intrinsic calibration parameters of the camera. Hence, the YOLO v2 object detector can be applied to a single search window centered at the predicted target projection on the image plane. The horizontal and vertical sizes of this search window, i.e., *d_u,tr_* and *d_v,tr_*, respectively, can be set by the user considering that their minimum value is 150 pixels (as the minimum size for the input of the YOLO v2 detector is defined by the size of the training dataset). The selection of larger values for *d_u,tr_* and *d_v,tr_* can be convenient when dealing with fast relative dynamics relatively to the frame rate and, especially, if the target UAV is flying at close range with respect to the tracker (thus occupying large portions of the FOV).

**Figure 9:**
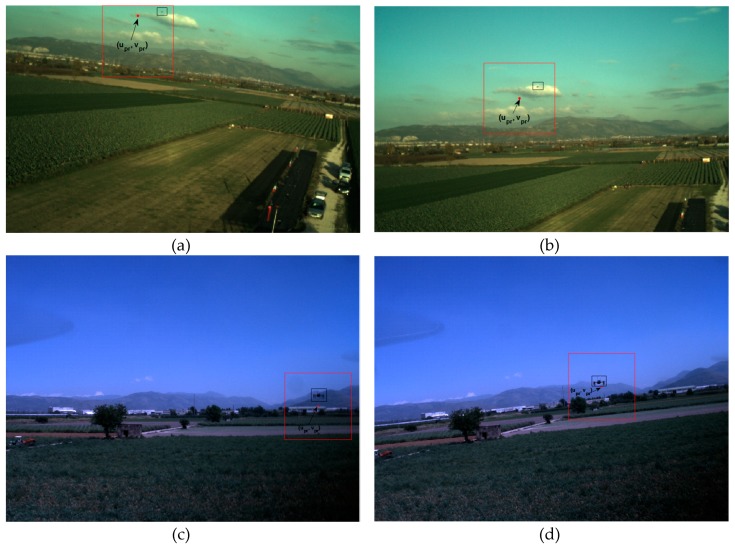
Examples of prediction of the target UAV projection on the image plane (highlighted by a red dot) carried out by the DL-based tracker. The search area is drawn as a red square. The target UAV is enclosed in a black box. (**a**,**b**) Far range scenario (target range ≈ 116 m); *d_u,tr_* = *d_v,tr_* = 150 pixels; prediction error ≈ 45 pixels. (**c**,**d**) Close range scenario (target range ≈ 20 m); *d_u,tr_* = *d_v,tr_* = 300 pixels; prediction error ≈ 20 pixels.

Clearly, this solution allows significantly reducing the number of computations with respect to the DL-based detector. Some examples of search area definition during the tracking process for flight tests characterized by different target, camera (detector and optics) and frame rate are shown in Figure 9. These examples are relative to extreme cases, i.e., the target-tracker relative dynamics is particularly aggressive relatively to the frame rate, thus causing a larger prediction error. The frames depicted in Figure 9a,b are acquired using a 752 × 480-pixel detector at 1 fps. Due to the small size of the target (far range scenario), *d_u,tr_* and *d_v,tr_* are set to 150 pixels. Instead, the frames depicted in Figure 9c,d are acquired using a 1600 × 1200-pixel detector at 7 fps. Due to the large size of the target (close range scenario), *d_u,tr_* and *d_v,tr_* are set to 300 pixels. Finally, it is possible to notice that if the prediction is too close to the borders of the image plane, the search window cannot be centered at that position, as in Figure 9a, but rather it must be adjusted to the size of the detector.

Once the YOLO v2 object detector is applied to the defined search window, as for the DL-based detector, the BB characterized by the maximum score is selected. The confirmation of this detection is again carried out applying a thresholding criterion but a different threshold (*τ_tr_*) with respect to the detection phase can be used. Clearly, also in this case, the confirmed BB is sent to the *BB refinement* block before estimating the target LOS and proceeding to the next frame.

## 4. Flight Test Campaign

A campaign of flight tests involving formations of two UAVs has been recently conducted to build a database of images suitable for training the deep neural network composing the YOLO v2 object detector exploited within the proposed visual-based architecture, as well as for assessing its performance for detection and tracking of cooperative UAVs. The database can be divided in two parts (A and B) obtained using different rotorcraft UAVs equipped with different cameras.

**Figure 10:**
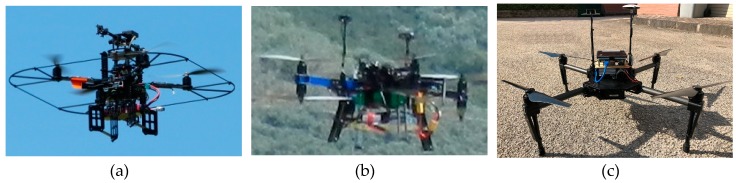
UAVs exploited for the flight test campaign. (**a**) Tracker UAV for database A: customized Pelican by Ascending Technologies. (**b**) Target UAV for database A: customized X8+ by 3D Robotics. (**c**) Target and tracker UAV for database B: customized M100 by DJI.

For Database A, the role of the tracker UAV is played by the Pelican^TM^ quadcopter depicted in Figure 10a. This UAV, produced by Ascending Technologies (http://www.asctec.de), is equipped with an autopilot characterized by a 1000 Hz update rate in the inertial guidance system, an onboard computer (AscTec Mastermind^TM^) featuring an Intel i7 processor, a GNSS receiver and a low-cost IMU unit based on MEMS sensors. This vehicle is customized by the installation of an additional GNSS receiver (LEA-6T produced by Ublox^TM^) and antenna to enable the acquisition of raw GNSS measurements (e.g., pseudoranges) not accessible from the AscTec autopilot, while a miniaturized CMOS camera (mvBlueFOX-MLC-200wc produced by Matrix Vision^TM^) with 752-by-480 pixel resolution is mounted on the top of the Pelican structure in frontal looking configuration for detection and tracking purposes. The camera is equipped with a 6.5-mm focal length optics, with a resulting IFOV of about 0.05° Both these auxiliary sensors are connected to the AscTec Mastermind via a USB link. The role of the target is played by a X8+ octocopter produced by 3D Robotics^TM^ (https://3dr.com/) depicted in Figure 10b. As in the case of the Pelican, the X8+ is customized by the installation of an auxiliary GNSS system, which is connected via USB to an embedded CPU (i.e., the Odroid XU4^TM^) for data acquisition and storage.

For Database B, the roles of tracker and target are played by two customized versions of the DJI M100^TM^ quadrotor depicted in Figure 10c. They are both equipped with an onboard computer (Intel NUC^TM^ with i7 CPU) and an additional GNSS single frequency receiver (uBlox LEA-M8T^TM^) with raw measurements capabilities and an auxiliary GNSS antenna. The tracker vehicle is also equipped with an off-the-shelf CMOS camera for vision-based detection and tracking purposes (Flea FL3-U3-20E4C-C produced by FLRI^TM^) characterized by 1600-by-1200 resolution in pixels and maximum frame rate of 59 fps. The camera is equipped with an 8-mm focal length optics, with a resulting IFOV of about 0.03°. Again, the auxiliary visual and GNSS sensors are connected to the Intel NUC via USB.

This flight test campaign is conducted using the approach of data acquisition for off-line processing. Specifically, an ad-hoc developed acquisition software is used to save all the sensor data with an accurate time-tag based on the CPU clock. This time-tag is associated with GNSS measurements, which include the GPS time, with very small latency. This solution has a key role to enable the use of a cooperative approach since it allows accurately synchronizing all data acquired on each flying vehicle.

Clearly, with regards to a real-time implementation of the proposed approach, a V2V communication link shall be foreseen to enable the exchange of navigation information required to estimate the target-tracker relative range and line of sight in ECEF or NED. However, the amount of this information (e.g., GNSS data) is limited to a few Kbits per second. Hence, this constraint can be easily met by standard V2V communication systems [65].

### 4.1. Database A

This database is composed of images collected during three Flight Tests (FT), that are indicated as FT1-A, FT2-A and FT3-A in the following discussion. The adopted testing strategy is aimed at assessing the capability of the proposed vision-based architecture to detect and track a cooperative vehicle flying at relatively-far range, i.e., when it occupies only a limited portion of the FOV. Indeed, the target-tracker relative distance which is computed as the difference between the synchronized GNSS position fixes provided by the receivers on board of the two vehicles, varies from a minimum of 53 m (the target occupies a rectangular region of about 15 × 10 pixels) to a maximum of 153 m (the target occupies very few pixels). Moreover, during each FT, a series of 360° turns is commanded to the tracker UAV to verify whether the proposed architecture can autonomously recognize when the target falls outside the FOV and to redetect it once the rotation is ended. Due to this testing strategy, the total number of collected images (*N*) can be divided in frames either containing (*N_IN_*) or not containing the target (*N_OUT_*). The acquisition software executed on board of the tracker UAV is designed so that the image acquisition is triggered by the reception of a GNSS package. This solution allows getting corresponding visual and positioning data with a negligible latency and an update rate of 1 Hz. Due to this choice, the capability of the camera to acquire and save images at higher frame rates (e.g., around 20–30 Hz) is not exploited. However, this solution allows testing the proposed vision-based architecture against more complex conditions for tracking since the target position in the FOV experiences a much faster dynamic. Main information about the FTs are synthetized in Table 1, where this latter phenomenon is quantitatively measured by the mean and standard deviation of the target displacement between consecutive frames (*∆u* and *∆v* for the horizontal and vertical direction, respectively), which is of the order of tens of pixels.

During all these flights, the local background (with respect to the position of the tracker in the FOV) is characterized by a significant variability in terms of color and clutter level due to the presence of mountains and vegetation. 

**Figure 11:**
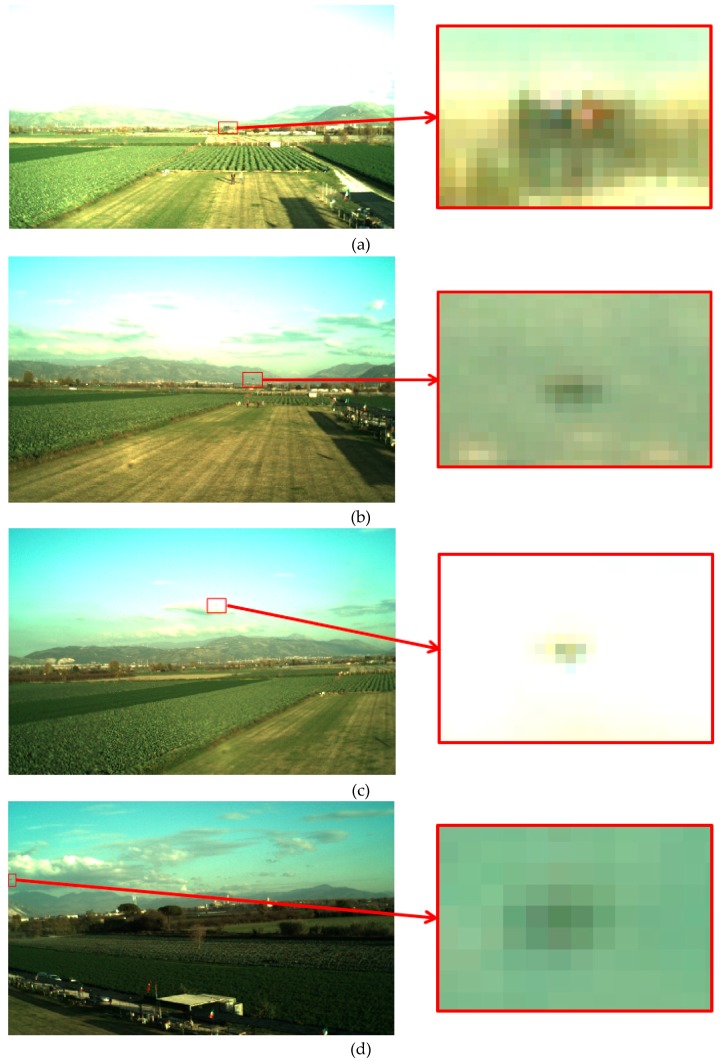
Example of images from FT3-A. The target (i.e., the X8+ octocopter) occupies a few pixels as highlighted by the zoom on the right side of each figure. (**a**,**b**). Target below the horizon. (**c**,**d**) Target above the horizon hindered by clouds.

Even when the target is above the horizon, the background conditions can be challenging when the target UAV is hindered by clouds or if most of the FOV is saturated due to sudden variation of the Sun illumination conditions. A small set of images with a zoom on the target is collected in Figure 11 to provide examples of the above-mentioned challenges to the effectiveness of the proposed approach.

### 4.2. Database B

This database is composed of images collected during two FTs, that will be indicated as FT1-B and FT2-B in the following. The former FT allows assessing the performance of the proposed vision-based architecture in a wider interval of ranges, i.e., from a minimum of 25 m to a maximum of 300 m. The capability to image the target UAV at longer distance with respect to the Database A is due to the better resolution of the camera on board of the tracker UAV (the IFOV is 0.03° instead of 0.05°). Instead, FT2-B is a coordinated flight at close range, i.e., the two UAVs move in the same direction while keeping the target-tracker distance in a short range of variation (from 17 m and 23 m). 

**Figure 12:**
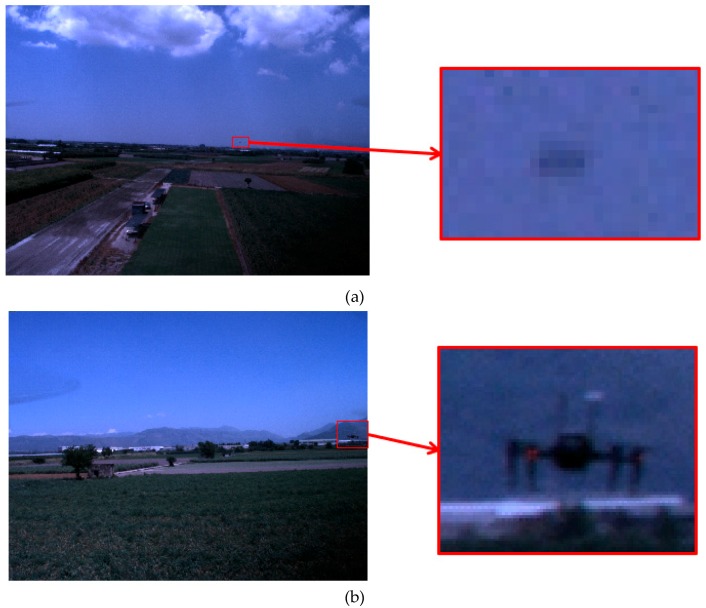
Example of images from FT1-B (**a**) and FT2-B (**b**).

A different data acquisition software is executed on board of the tracker UAV for these FTs, which allows collecting images with an update rate of 7 Hz. It is developed in Robot Operating System (ROS) and it includes a custom ROS node coded in C++ to process GNSS receiver data and to get an accurate time tag based on GPS time and the CPU clock. Due to the higher frame rate, the frame-to-frame displacement on the image plane is smoother than for Database A. Like in the previous subsection, some statistics to summarize the content of this database are collected in Table 2. A small set of images from the two FTs with a zoom on the target is collected in Figure 12 to provide examples of the target appearance.

## 5. Results

The YOLO v2 object detector is trained using a sub-set of 650 images selected from FT1-A and FT2-A. As anticipated in Section 3.1.2, these images are randomly cropped around the target with 150-by-150-pixel windows and labelled by a human operator to obtain the ground truth (supervised approach). The resulting neural network is first used within the *BB detection* block to test the performance of the proposed architecture on FT3-A. Then, the generalizing capabilities of the network are evaluated by testing the proposed architecture with images obtained during FT1-B and FT2-B which are different in terms of target UAV, camera installed on board the tracker and interval of target-tracker relative distance. Also, these flights are carried out on a different flight field and in a different period of the year.

Before entering the details of the achieved results, it is necessary to provide a concise definition of the performance parameters which are adopted within the next sub-sections. Specifically, the detection and tracking performance is analyzed in terms of four performance parameters.

- *Percentage of Correct Detections* (CD), i.e., the ratio between the number of frames in which the target is correctly declared to be either inside the image plane (less than 10-pixel error both horizontally and vertically) or outside the image plane and the total number of frames.
- *Percentage of Wrong Detections* (WD), i.e., the ratio between the number of frames in which the target is correctly declared to be inside the image plane but the error is larger than 10 pixels either horizontally or vertically, and the total number of frames.
- *Percentage of Missed Detections* (MD), i.e., the ratio between the number of frames in which the target is wrongly declared to be outside the image plane (it is not detected even if it is present in the image) and the total number of frames.
- *Percentage of False Alarms* (FA), i.e., the ratio between the number of frames in which the target is wrongly declared to be inside the image plane (it is detected even if it is not present in the image) and the total number of frames.

### 5.1. Detector Performance on FT3-A 

**Figure 13:**
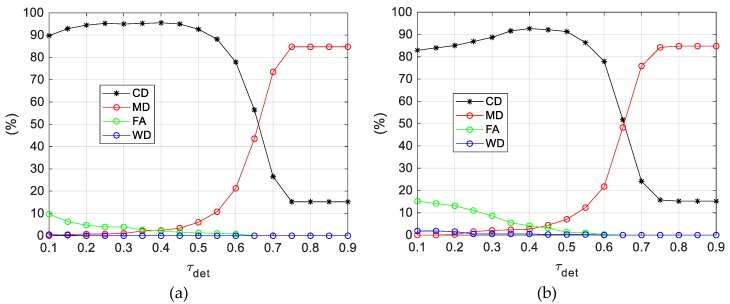
DL-based detector performance as a function of *τ_det_*. FT3-A composed of 381 frames. (**a**) *Target UAV prediction* enabled (*d_u_* = 100 pixels, *N_w_* = 7 search windows). (**b**) *Target UAV prediction* disabled (*d_u_* = *d_v_* = 100 pixels, *N_w_* = 28 search windows).

A detailed analysis of the performance of the first step of the proposed visual-based architecture is carried out. Specifically, the DL-based detector is applied to each image acquired during FT3-A and the previously-defined performance metric are evaluated as a function of *τ_det_*, i.e., the minimum value of the maximum score among the search windows (*S_max_*) for which the detected BB is confirmed. These results are collected in Figure 13a. To highlight the advantages gained by exploiting the navigation data of both the tracker and the target to limit the search area, the performance metrics are also evaluated applying the DL-based detector without the *Target UAV prediction* block. In this case the YOLO v2 object detector is applied to a set of squared search windows composed of 150 × 150 pixels defined to cover the entire image plane. These results are collected in Figure 13b. As explained in Section 3.1.2, *d_u_* is set to 100 pixels to obtain a partial overlap between consecutive windows. Clearly, a similar parameter can be defined in the vertical direction for the full-image analysis which is also set to 100 pixels.

By enabling the *Target UAV prediction* block, the average computational time gets 75% lower than for the full-image analysis. Besides this expected advantage in terms of computational efficiency due to the reduced number of search windows, the results above show that the use of target and tracker navigation data allows improving the detector performance for any considered metric and for any value of *τ_det_*. Clearly, the most critical advantage is given by the significant reduction in FA, i.e., 53% on average in the *τ_det_*-interval (0.10, 0.60). This phenomenon can be intuitively explained considering that the possibility that the YOLO v2 object detector provides a BB with a relatively-high score (containing something with the same appearance of the target UAV, like a bush on ground or a bird in the sky) increases if the target search is carried out in the full image plane, and especially if a large portion of the scene is below the horizon.

Thus, focusing on the performance level achieved by the full DL-based detector, as depicted in Figure 13a, some interesting considerations can be made:

- WD is always 0% (except for a couple of wrong detections occurring if *τ_det_* is set below 0.20), which implies that, when the target is inside the FOV, it is always detected, and its LOS is estimated with a relatively-high accuracy level.
- CD and MD reach an asymptotic value (15.2% and 84.8%, respectively) when *τ_det_* gets larger than 0.70. This occurs since the selection of large values of *τ_det_* prevents false alarms for the 58 images (see *N_OUT_* in Table 1) where the target is outside the FOV (or is not visible).
- The best performance in term of CD (95.5%) is obtained setting *τ_det_* to 0.40. However, this choice leads to a residual FA of 2.1%. As *τ_det_* increases, FA goes to 0% but MD also increases to the detriment of the number of correct detections. In general, the trade-off in the choice of *τ_det_*, i.e., the problem of finding the best compromise between CD, FA and MD, depends on the mission task. For instance, cooperative UAVs can be used to improve navigation performance in GNSS-challenging areas [13]. In this case it is more important to limit the occurrence of false alarms, thus choosing larger values of *τ_det_*.

Another interesting analysis regards the effectiveness of the *BB refinement* block described in Section 3.1.3 aiming at improving the accuracy achieved in the estimation of the target projection on the image plane, and consequently, on the corresponding LOS. This accuracy is evaluated as the error between the true and estimated azimuth (*Az*) and elevation (*El*) angles which can be obtained from the true and estimated target LOS, respectively. The relations between the target LOS in CRF (*ρ_c,los_*) and the two angles are given by Equations (3) and (4):

(3) $$Az=\tan^{-1}\left(\frac{\rho_{x}}{\rho_{z}}\right)$$

(4) $$El=\tan^{-1}\left(-\frac{\rho_{y}}{\rho_{z}}\cos\left(Az\right)\right)$$

where *ρ_x_*, *ρ_y_* and *ρ_z_* are the components of *ρ_c,los_* in CRF. A statistical analysis of the azimuth and elevation accuracy achieved either enabling the *BB refinement* block or not, and setting *τ_det_* to 0.40, is presented in Table 3.

The introduction of the *BB refinement* block determines an improvement in all the statistical parameters both for the azimuth and elevation angles. In particular, the uncertainty (std) is reduced of around 50% in *Az*, and 68% in *El*, and a slight reduction is also observed in the mean error. Even including this systematic error, the rms value shows that the use of the *BB refinement* block allows achieving an accuracy of the order of the camera IFOV (0.05°) in both *Az* and *El*. Further error reduction is hindered by the uncertainty of the supervised approach used to mark the ground truth, especially at far target-tracker range, when the target is extremely small and blurred on the image plane. Finally, a comparison is presented in Table 4 between the best detection performance achieved on this FT using the proposed DL-based approach (*τ_det_* = 0.40) against a state-of-the-art technique based on standard machine vision tools (i.e., template matching and morphological filtering) proposed by the authors in [48].

### 5.2. Detector and Tracker Performance on FT3-A 

The performance achieved by the full visual-based architecture proposed for detection and tracking of cooperative UAVs is now evaluated, considering the effects determined by the choice of the two main setting parameters, i.e., *τ_det_* and *τ_tr_*. Based on the results presented in the previous section in Figure 13a, *τ_det_* is first set to 0.40 (which provided the best performance in terms of CD) while *τ_tr_* is varied between 0.40 and 0.10. 

The choice to set *τ_tr_* to values less than or equal to *τ_det_* is in line with the idea implemented by the DL-based tracker to limit the target search to a single window centered at the predicted target projection where the confidence to find the target is higher than in other regions of the image plane. The result of these tests obtained setting *d_u,tr_* and *d_v,tr_* to 150 pixels, are collected in Table 5.

The advantage of using the full detection and tracking architecture with respect to the DL-based detector only, is twofold. Clearly, it is mainly related to the improvement in the computational efficiency, since the YOLO v2 object detector is applied to a single search window. However, it is also possible to reduce the number of false alarms (setting *τ_tr_* to 0.40) or to optimize the ratio between CD and MD (lowering *τ_tr_* to 0.20).

To better highlight the advantage in terms of the performance metrics, gained using the DL-based tracker, and, consequently, optimizing the performance of the visual-based architecture, it is convenient to increase the value of *τ_det_*. Indeed, it is reasonable to have less confidence in the target projection identified at the detection stage due to the lower amount of available a-priori information. Looking again at the results in Figure 13a, *τ_det_* is now set to 0.65, which is the minimum value which allows nullifying FA (at the expense of an increase in MD). Instead, *τ_tr_* is varied from 0.65 down to 0.20 (lower values are not considered as they tend to produce more false alarms without improvement in CD). The result of these tests (setting again *d_u,tr_* and *d_v,tr_* to 150 pixels), are collected in Table 6.

Thanks to the selection of a larger detection threshold, the absence of false alarms is confirmed even if the tracking threshold is significantly reduced (down to 0.20), which simultaneously allows reaching a peak level for CD. Indeed, the remaining missed detections correspond to image frames in which the DL-based detector correctly determines the target position, but the score is lower than 0.65. With this configuration for the setting parameters (*τ_det_* = 0.65 and *τ_tr_* = 0.20), the accuracy level in the estimated LOS of the target is characterized by a rms error of 0.050° and 0.060° in azimuth and elevation, respectively.

### 5.3. Detector and Tracker Performance on FT1-B and FT2-B 

The proposed algorithmic architecture for visual-based detection and tracking of cooperative UAV is now tested using a dataset of images acquired by a different tracker UAV equipped with a camera characterized by a better spatial resolution (1600 × 1200 pixels) at a higher frame-rate (see Section 4.2 for details). This latter aspect makes more effective the strategy of exploiting target and tracker navigation data to predict the target projection on the image plane during both the detection and tracking phases. Since also the target UAV is different from the previously-analyzed FTs, these tests allow preliminary assessing the generalizing capability of the YOLO v2 object detection system trained for the *BB detection* block within the DL-based detector and tracker.

Starting from FT1-B which presents more challenging conditions due to the extremely wide variation of the target-tracker range (from 25 m to 300 m), the performance metrics are evaluated considering different values of *τ_det_* and *τ_tr_*. First, *τ_det_* is set to 0.50 and *τ_tr_* is varied from 0.50 and 0.075. The results of these tests obtained setting *d_u,det_* to 100 (*N_w_* = 15), *d_u,tr_* and *d_v,tr_* to 150 pixels, are collected in Table 7.

**Figure 14:**
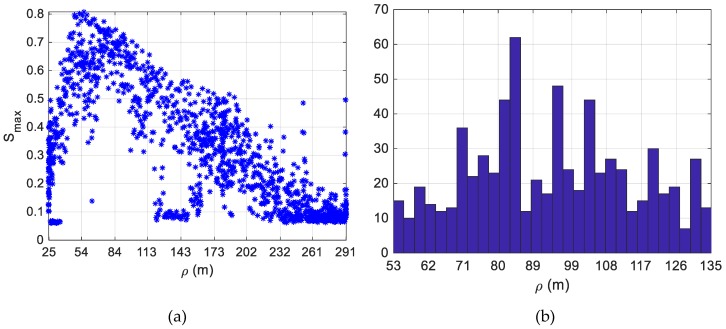
(**a**) Detection and tracking test on FT1-B (1330 images). Distribution of *S_max_* as a function of the target-tracker relative distance. (**b**) Histogram providing the distribution of the target-tracker range characterizing the 650 images selected from FT1-A and FT2-A.

Though this configuration of the setting parameters produces very low values of FA (up to 4 false alarms if *τ_tr_* is 0.075), it limits the number of correct detections. This phenomenon can be explained considering that, due to the difference in the flight-test scenario, and, consequently, in the images collected during FT1-B with respect to the training dataset (obtained from FT1-A and FT2-A), the average score characterizing the BBs provided by the YOLO v2 object detector is lower than in the previous tests carried out on FT3-A. Specifically, there is a correlation between the target-tracker relative distance and the value of *S_max_*, as shown in Figure 14a. As it could be expected, larger values of *S_max_* (up to 0.8) are obtained when the target-chaser relative distance is in the interval covered by the training dataset (53–135 m). A histogram providing the distribution of the range characterizing the training images can be found in Figure 14b.

By looking at the significant increase in CD obtained by varying *τ_tr_* from 0.50 (28.27%) to 0.075 (56%), it is clear that lower values of *τ_det_* must be selected to try further increasing the number of correct detections. Hence, the result of additional tests obtained setting *d_u,det_* to 100 (*N_w_* = 15), *d_u,tr_* and *d_v,tr_* to 150 pixels, are collected in Table 8.

**Figure 15:**
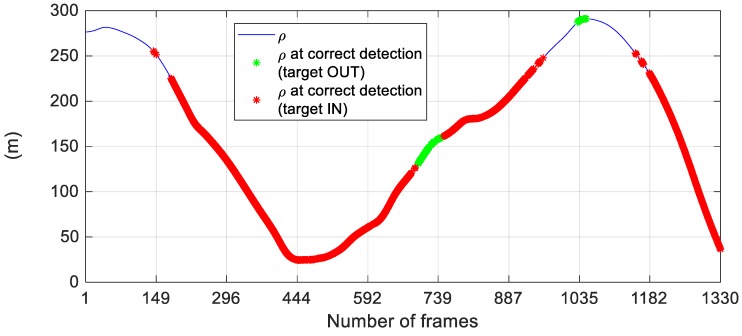
Variation of the target-chaser relative distance (blue line) during the FT1-B (1330 images). The DL-based detector and tracker are applied setting *τ_det_* to 0.20 and *τ_tr_* to 0.075. The frames where the algorithmic architecture provides correct detections are highlighted with red (target inside the FOV) and green (target outside the FOV) stars.

By setting *τ_det_* to 0.20 and *τ_tr_* to 0.075, it is possible to get a peak value of CD (67.59%) while keeping the number of false alarms and wrong detections below 40 out of 1330 tested frames. Looking at the still relatively large value of MD, the achieved performance could appear poor if compared with the results shown regarding FT3-A. However, the missed detections are related exclusively to a limit in the detection range. To clarify this point, Figure 15 shows the variation of the target-tracker relative distance during FT1-B, while highlighting the instances of correct detection. 

The proposed architecture can detect and track the target during the entire flight test. A relatively-large number of missed detections (which determines the percentage values of MD in Table 8) occurs only when the target-tracker relative distance gets larger than 225 m, which is well beyond the maximum target-tracker range characterizing the set of training images (≈135 m). Indeed, at such far range, the target not only occupies very few pixels on the image plane, but it is also blurred (its appearance is characterized by very low contrast with respect to the local background even above the horizon). Finally, Table 9 presents a statistical analysis of the accuracy level attained in the estimated LOS of the target for different values of the setting parameters.

These results show that the best setting in terms of CD (*τ_det_* = 0.20 and *τ_tr_* = 0.075) leads to a rms error slightly larger (lower) than twice the camera IFOV (0.03°) for the *Az* (*El*) angle. Instead, if the best setting in terms of FA is selected (*τ_det_* = 0.50 and *τ_tr_* = 0.15), the rms error is around 0.04° both horizontally and vertically. This can be explained considering that this latter configuration of the setting parameters allows removing a larger number of correct detections but characterized by lower values of *S_max_*, and, consequently, by a lower overlap with the real target position on the image plane.

An additional test is now carried out applying the proposed detection and tracking architecture to the images collected during FT2-B which is a coordinated flight at close range, i.e., the target-tracker relative distance varies in the interval (17 m, 23 m). During this image sequence the target is always inside the FOV so false alarms cannot occur. Since the target occupies a larger portion of the image plane with respect to the previously analyzed flight tests, the size of the search window is increased becoming a square of 300 × 300 pixels. This solution is adopted to avoid risking that the target is partially cut out of the search area either during the detection or the tracking process. Based on the results on FT1-B, *τ_tr_* is set to a low value (0.10) while different values of *τ_det_* are considered. Results of these tests are collected in Table 10.

The results show that it is possible to significantly increase CD by reducing the value of *τ_det_* down to 0.20. This solution allows correctly detecting and tracking the target during almost the entire flight, without causing an increase in the number of wrong detections However, the choice of accepting detections characterized by a low value of *S_max_* leads to a slight worsening of the accuracy in the estimated LOS of the target. Indeed, if *τ_det_* and *τ_tr_* are set to 0.20 and 0.10 respectively, the rms error is around 0.08° in both *Az* and *El*.

## 6. Conclusions

This paper proposed an original algorithmic architecture for visual-based detection and tracking of cooperative UAVs. This architecture relies on a state-of-the-art Deep Learning neural network (YOLO v2) as main processing block, but it exploits navigation data of both the tracker and target UAV (also available thanks to the cooperative nature of the formation) to obtain a reliable prediction of the position of the target projection on the image plane, thus limiting the area to which the object detector must be applied. This strategy allows not only the computational time to be reduced, but it also extremely limits the number of false alarms which tend to occur when the target search is carried out in the entire image plane. Another innovative point is the development of an image processing technique to refine the position of the bounding box detected by YOLO, thus increasing the angular accuracy in the estimate of the target line-of-sight.

An experimental campaign of flight tests involving formations of two multirotor UAVs was carried out to build a database of images to both train the neural network and assess the performance of the proposed architecture. The flight tests were carried out with different UAVs as both tracker and target vehicles and using cameras with different resolution. The developed detection and tracking algorithms were tested over multiple datasets, thus being able to analyze the reliability and robustness against challenging conditions. In particular, the testing datasets were characterized by wide variability of the target appearance in terms of clutter level in the background, illumination condition and scale. Regarding this latter aspect, the generalizing capability of the trained neural network was evaluated due to the extremely wide variability of the target-tracker relative distance (from 17 m to 300 m) in the analyzed flight tests. The proposed architecture showed satisfying performance (more than 90% of correct detections) in all the analyzed cases while being able to estimate the target line-of-sight with an accuracy of the order of the camera IFOV (from 0.04° to 0.08° in the different cases).

Further investigations are still needed to improve the capability to correctly identify the target at far range below the horizon, where the target appearance may have very low contrast with respect to the local background. In fact, most of the wrong detections and false alarms noted analyzing the detection and tracking performance of the proposed architecture over the two datasets, occur below the horizon where the local background is particularly cluttered. Indeed, the closer the background is with respect to the camera, the lower the local contrast produced by the target gets and, consequently, the more difficult the detection process becomes. This problem could be addressed by training different DL-based neural network to operate above and below the horizon, respectively. Clearly, this solution would foresee the implementation of a reliable processing strategy to extract the horizon in order to decide which detector is more convenient depending on the position of the target prediction. Another open point for investigation regards the possibility to take advantage of the knowledge of the target-tracker relative distance also during the training process. This would allow entrusting the detection task to different neural networks each one purposely trained in a different interval of range, with the aim of increasing both reliability and accuracy. Indeed, the results presented in terms of the generalization capability of the neural network trained in this work, show that it is challenging to apply the same DL-based detector when the target-tracker distance gets too large or short with respect to the interval of ranges characterizing the training dataset. Overall, the possibility to investigate these open points leads to the need to generate a larger database of visual data by conducting additional flight tests. Finally, it is worth outlining that different DL-based object detector, other than YOLO v2, could be trained and tested within the proposed architecture without the need of further modifying the processing strategies. 

## Figures and Tables

**Table 1:** Statistics of the FTs for database A.

FT	*N*	*N_IN_*	*N_OUT_*	*ρ* (m)		*∆u*/*∆v* (pixel)	
				Mean	Std	Mean	Std
**1**	398	376	22	84.2	17.1	38/24	59/26
**2**	361	319	42	105.9	19.5	30/26	50/26
**3**	381	323	58	120.2	19.5	33/21	42/21

**Table 2:** Statistics of the FTs for database B.

FT	*N*	*N_IN_*	*N_OUT_*	*ρ* (m)		*∆u*/*∆v* (pixel)	
				Mean	Std	Mean	Std
**1**	1330	1275	75	168.9	86.5	4/2	9/4
**2**	735	735	0	20.5	1.7	4/7	6/9

**Table 3:** Statistical analysis of the accuracy in the estimated target LOS. DL-based detector applied on the images (381) from FT3-A (*τ_det_* = 0.40).

	*BB Refinement Enabled*		*BB Refinement Not Enabled*	
	*Az Error (°)*	*El Error (°)*	*Az Error (°)*	*El Error (°)*
**Mean**	−0.046	0.057	−0.049	0.063
**Std**	0.023	0.016	0.046	0.051
**Rms**	0.051	0.059	0.067	0.081

**Table 4:** Comparison between the detector proposed in this paper (DL-based) and a state-of-the-art approach [48], based on template matching and morphological filtering. FT3-A (381 images).

Detector	*CD*	*MD*	*FA + WD*
**DL-based**	95.5%	2.4%	2.1%
**[48]**	78%	18%	4%

**Table 5:** DL-based detector vs. DL-based detector and tracker performance as a function of *τ_tr_* (*τ_det_* = 0.40). FT3-A composed of 381 frames.

	*τ_det_*	*τ_tr_*	MD	FA	CD	WD
**DL-based detector**	0.40	/	2.4%	2.1%	95.5%	0.0%
**DL-based detector and tracker**	0.40	0.40	3.2%	1.8%	95.0%	0.0%
	0.40	0.30	1.8%	2.1%	96.1%	0.0%
	0.40	0.20	1.3%	2.4%	96.3%	0.0%
	0.40	0.10	0.5%	3.7%	95.5%	0.3%

**Table 6:** DL-based detector vs. DL-based detector and tracker performance as a function of *τ_tr_* (*τ_det_* = 0.65). FT3-A composed of 381 frames.

	*τ_det_*	*τ_tr_*	MD	FA	CD	WD
**DL-based detector**	0.65	/	43.6%	0%	56.4%	0%
**DL-based detector and tracker**	0.65	0.65	40.4%	0%	59.6%	0%
	0.65	0.40	11.0%	0%	89.0%	0%
	0.65	0.20	7.35%	0%	92.65%	0%

**Table 7:** DL-based detector and tracker performance as a function of *τ_tr_* (*τ_det_* = 0.50). FT1-B composed of 1330 frames.

	*τ_det_*	*τ_tr_*	MD	FA	CD	WD
**DL-based detector and tracker**	0.50	0.50	71.65%	0.08%	28.27%	0%
	0.50	0.30	61.73%	0.15%	38.12%	0%
	0.50	0.15	58.2%	0.15%	41.65%	0%
	0.50	0.075	42.8%	0.3%	56%	0.9%

**Table 8:** DL-based detector and tracker performance as a function of *τ_tr_* (*τ_det_* = 0.20). FT1-B composed of 1330 frames.

	*τ_det_*	*τ_tr_*	MD	FA	CD	WD
**DL-based detector and tracker**	0.20	0.20	39.62%	1.05%	57.59%	1.73%
	0.20	0.15	36.54%	1.05%	60.68%	1.73%
	0.20	0.10	31.65%	1.20%	65.64%	1.50%
	0.20	0.075	29.55%	1.43%	67.59%	1.43%

**Table 9:** Statistical analysis of the accuracy in the estimated target LOS. DL-based detector and tracker applied on the images (1330) from FT1-B.

	*τ_det_ = 0.20 and τ_tr_ = 0.075*		*τ_det_ = 0.50 and τ_tr_ = 0.15*	
	*Az Error (°)*	*El Error (°)*	*Az Error (°)*	*El Error (°)*
**Mean**	−0.016	0.040	−0.025	0.036
**Std**	0.062	0.034	0.035	0.023
**Rms**	0.064	0.053	0.043	0.043

**Table 10:** DL-based detector and tracker performance as a function of *τ_det_* (*τ_tr_* = 0.10). FT2-B composed of 735 frames.

	*τ_det_*	MD	CD	WD
**DL-based detector and tracker**	0.50	23.3%	75.9%	0.8%
	0.30	5.3%	93.6%	1.1%
	0.20	4.9%	94%	1.1%
